# Endocrine cell micronests in an ovarian mucinous borderline tumor: a potential diagnostic pitfall for microinvasion

**DOI:** 10.1186/s13000-022-01217-4

**Published:** 2022-04-14

**Authors:** Katrina Collins, Sheila Segura, Michael Hwang

**Affiliations:** Department of Pathology, Indiana University, 350 W 11th Street, IN 46202 Indianapolis, USA

**Keywords:** Endocrine micronodules, Microinvasion, Endocrine cell micronests, Ovary, Mucinous tumors

## Abstract

The occurrence of endocrine cell micronests in ovarian tumors is rarely reported. To our knowledge, there are only three prior cases reported to date: one occurring in an ovarian mucinous cystadenoma, one in an ovarian mucinous cystadenofibroma, and another in an ovarian mucinous carcinoma with a predominant borderline component. This is a 27-year-old woman that presented with a one-month history of abdominal pain and fullness. Imaging studies revealed a large multiloculated cystic and solid mass measuring 23 cm occupying the majority of the pelvis and abdomen concerning for a primary ovarian malignancy. The patient underwent a right salpingo-oophorectomy with appendectomy. Histologic sections from the ovary showed a multiloculated, cystic and focally solid mass lined by gastrointestinal-type mucinous epithelium with variable degrees of proliferation accounting for greater than 10% of the tumor. In addition to the mucinous epithelial component, there were several foci of bland, monotonous epithelioid cells arranged in solid nests with focal tubular/acinar formation within the fibrous septa and mucinous epithelium. Immunohistochemical studies showed that these cells were positive for cytokeratin, EMA, and synaptophysin, while negative for inhibin. The Ki-67 proliferation index was low (<1%). The presence of endocrine cell nests associated with an ovarian mucinous neoplasm is a rare phenomenon. Whether this represents preservation of endocrine cells in the context of epithelial degeneration or an independent neoplastic component is unclear. Progression related to this endocrine cell proliferation is unlikely and the recognition of this phenomenon holds more diagnostic value than prognostic significance, as it could be confused with microinvasion or sex cord stromal elements.

## Introduction

Mucinous tumors represent 10-15% of primary ovarian neoplasms and can be divided into three groups based on malignant behavior: benign, borderline, or malignant histologic variants [1]. Among benign ovarian tumors, mucinous cystadenomas and mucinous adenofibromas account for approximately 75% of cases. The second most common, borderline ovarian tumors or atypical proliferative ovarian tumors, account for approximately 15-20%. The remaining 5% of cases represent invasive primary ovarian mucinous carcinomas [1, 2]. In addition to gastrointestinal-type mucinous epithelium, the presence of endocrine cells in ovarian mucinous neoplasms has been recognized for decades [3–6]. The endocrine cells are frequently inconspicuous when they are distributed as scattered single cells in the mucinous glands on routine hematoxylin and eosin-stained (H&E) sections, but can be highlighted by use of special stains or immunohistochemistry. Though infrequent, endocrine cell proliferation can be found within the epithelium and stroma and can give rise to rare cases of mixed ovarian tumors composed of a combination of epithelial and endocrine elements [7–10].

Herein, we describe a case of ovarian mucinous borderline tumor with foci of microscopic endocrine cell nests in the stroma and adjacent mucinous epithelium. The endocrine cells are predominantly distributed in reactive stroma adjacent to glandular elements in various stages of degeneration. This histologic observation lends support to the theory that endocrine cell micronests are preserved endocrine cells in the context of glandular degeneration [8, 10]. The awareness of this phenomenon is helpful in diagnosis as endocrine cell micronests may show overlapping morphologic features with microinvasion or sex cord-stromal elements.

## Case report

**Fig. 1:**
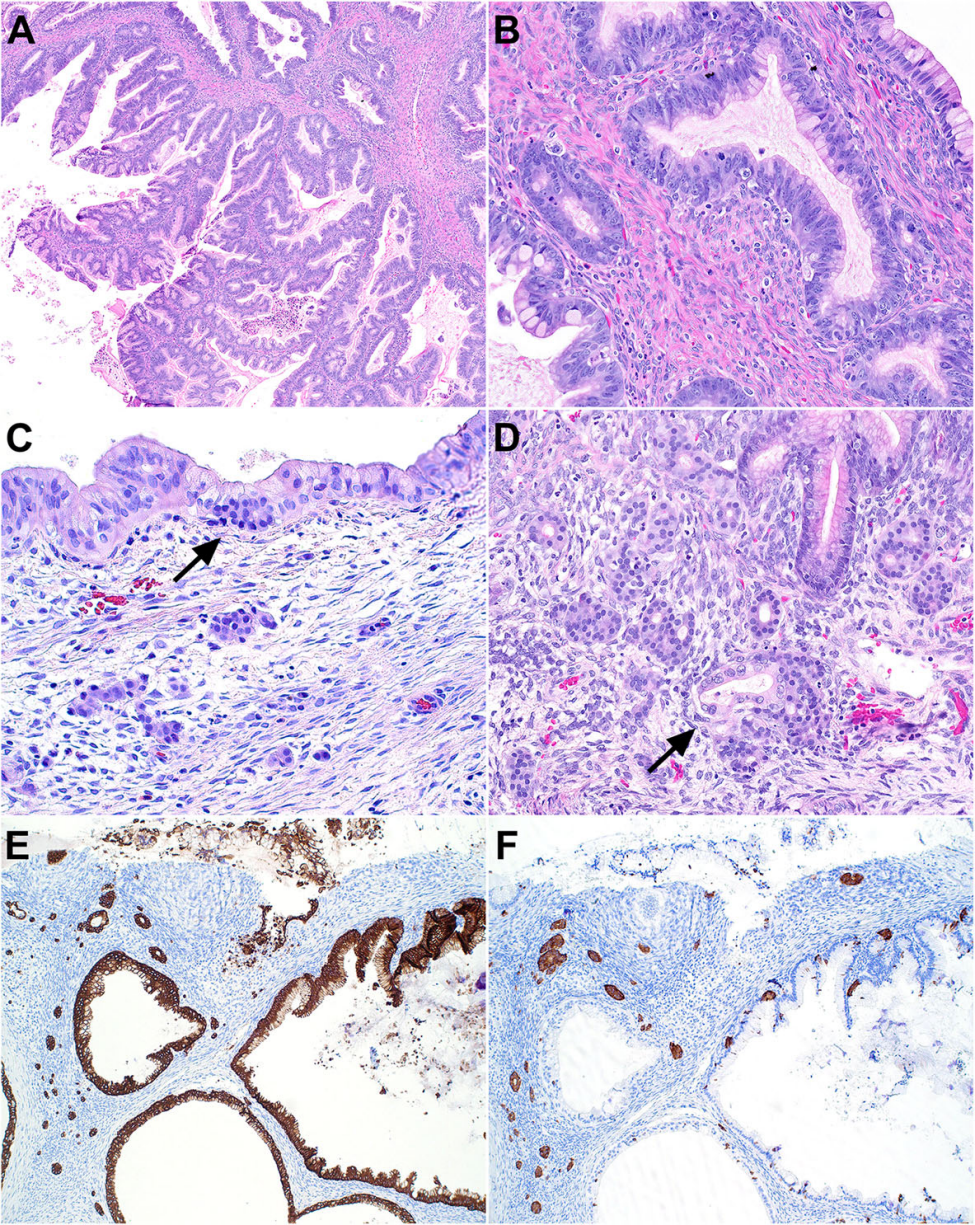
**A**, The cysts are lined by gastrointestinal-type mucinous epithelium with variable degrees of epithelial proliferation. **B**, Higher magnification demonstrating mucinous epithelium with goblet cells and nuclear atypia. **C**, There are several foci of bland, monotonous epithelioid cells arranged in solid nests or tubular/acinar architecture in reactive stroma adjacent to mucinous epithelium. The bland epithelioid cells can also be seen in adjacent mucinous glands (arrow). **D**, Florid stromal micronests are seen focally and cells emanating from a mucinous gland are present (arrow). **E**, Pancytokeratin (AE1/AE3) immunostaining highlights both mucinous epithelium and the stromal micronests. **F**, Synaptophysin immunostaining highlights the endocrine cell micronests in the stroma as well as intraepithelial neuroendocrine cells in adjacent mucinous glands

**Fig. 2:**
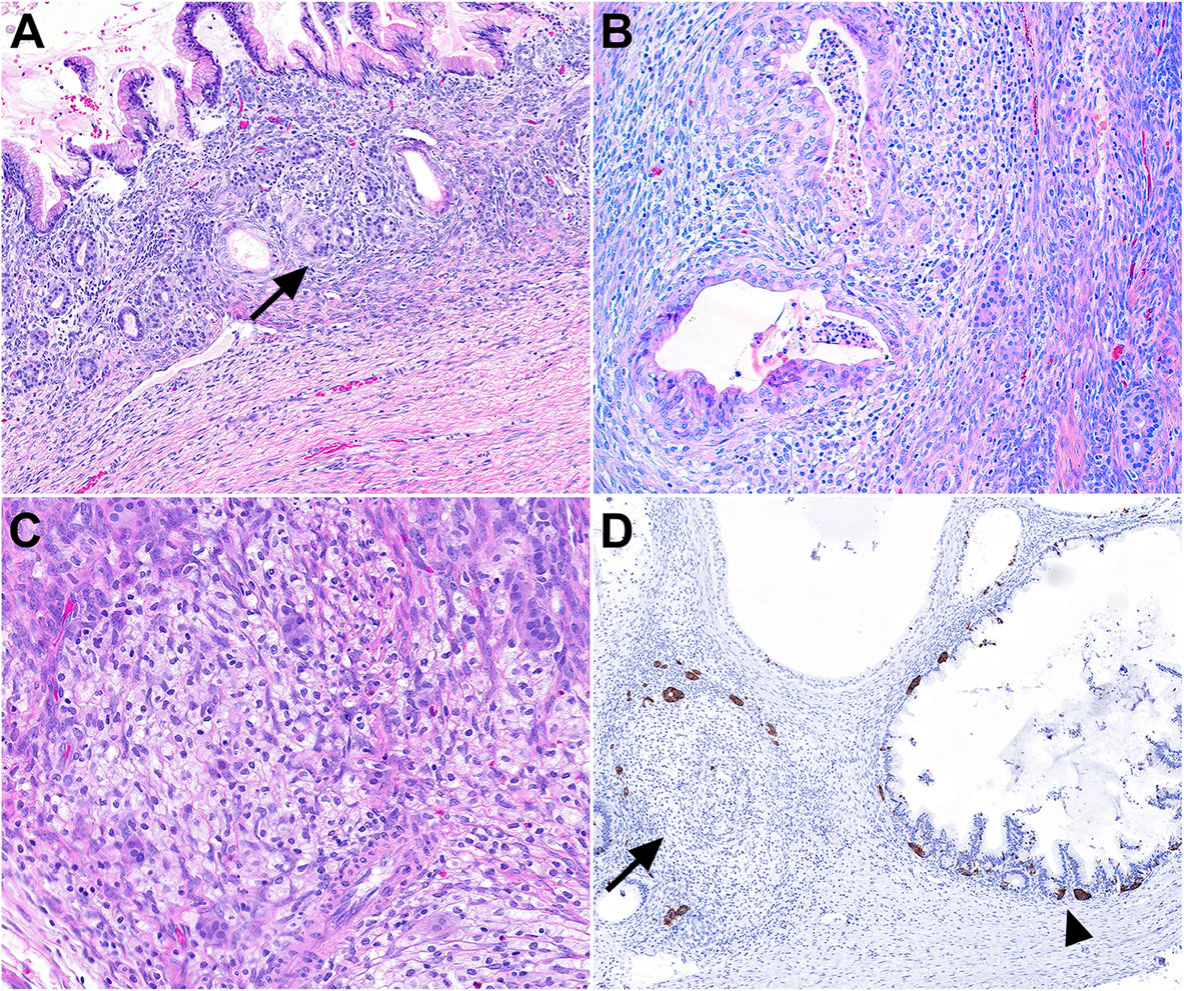
**A**, Endocrine cell micronests are seen in stroma adjacent to the mucinous cysts and their outpouching glands (arrow). **B**, Mucinous glands in early stage of degeneration with intraluminal neutrophils and stroma with reactive changes, histiocytes, and endocrine cell micronests. **C**, Round to ovoid shaped histiocytic infiltrates with endocrine cell micronests at the periphery, representing the late-stage glandular degeneration with preserved endocrine cells. **D.** Synaptophysin immunostaining highlights stromal endocrine cell micronests and intraepithelial neuroendocrine cells. A completely degenerated mucinous cyst with preserved neuroendocrine cells (arrow) is seen adjacent to a relatively intact mucinous cyst demonstrating intraepithelial neuroendocrine cells with variable degrees of proliferation (arrowhead)

The patient is a 27-year-old woman with no significant past medical history that presented with a one-month history of abdominal pain and fullness. Computed tomography (CT) scan revealed a complex, cystic right ovarian mass with internal septations and solid component occupying a majority of the pelvis and abdomen with prominent pelvic sidewall adenopathy. Preoperative serum CA- 125 was elevated at 174.9 U/mL (reference range: 0-35 U/mL). The patient underwent right salpingo-oophorectomy with appendectomy. The ovarian tumor measured 23 cm and weighed 2,890 g with a smooth and glistening intact capsule and an attached fimbriated fallopian tube. Sectioning of the mass revealed a multiloculated, cystic mass containing clear, watery serous fluid and thin mucoid material. The cysts were thin-walled with predominantly a smooth lining and focal solid growth (accounting for approximately 15-20%). On histologic examination, the cysts were lined by gastrointestinal-type mucinous epithelium with variable degrees of proliferation accounting for greater than 10% of the tumor (Fig. 1A and B). In addition to the mucinous epithelial component, there were several foci of bland, monotonous epithelioid cells arranged in solid nests with focal tubular/acinar arrangement scattered within the stroma and adjacent to and intermingled with mucinous epithelium (Fig. 1C and D). These epithelioid cells were generally uniform with bland, round nuclei, fine chromatin, and arranged in linear or micronodular clusters of at least 5 cells. No cytologic atypia or mitotic activity was identified. No teratomatous elements were identified despite extensive sampling of the tumor. Immunohistochemical studies showed that the epithelioid cells were positive for cytokeratin and EMA, while negative for inhibin (Fig. 1E and F). Synaptophysin highlighted individual cells and small nests of endocrine cells in adjacent mucinous glands. The Ki-67 proliferation index was low (<1%). The endocrine cell proliferations were focally present and often associated with outpouchings from large mucinous cysts (Fig. 2A), frequently noted adjacent to mucinous glands with degeneration (Fig. 2B and C), and associated with foamy histiocytes in areas with completely obliterated mucinous glands were occasionally seen (Fig. 2D).

## Discussion

Endocrine cells can be found in multiple organs throughout the body, but largely in the lungs, pancreas, and gastrointestinal tract. They may be seen as an inconsequential (minor) component of epithelial tumors or as a larger proportion of epithelial, germ cell, or sex cord stromal cell tumors. The presence of endocrine cell micronests associated with an ovarian mucinous neoplasm is a rare phenomenon that might cause diagnostic confusion when encountered. Whether this represents preservation of endocrine cells in the context of epithelial degeneration or an independent neoplastic component is unclear. Progression related to this endocrine cell proliferation is unlikely and the recognition of this phenomenon holds more diagnostic value than prognostic significance.

To our knowledge only 4 cases, including the present case, have been previously reported in mucinous neoplasia of the ovary (Table 1). In the first report, in addition to increased intraglandular endocrine cells, multiple foci of isolated endocrine cells and small nests were observed in fibrous stroma surrounding mucinous glands. This was thought to represent diffuse endocrine cell hyperplasia within a mucinous cystadenofibroma [7]. Similarly, in the second report, multifocal stromal endocrine cell nests were associated with increased intraglandular endocrine cells in the borderline component of mucinous carcinoma [8]. In the third report, the stroma showed multiple nests of endocrine cells with occasional follicle-like structures and was suggested to represent preservation of endocrine cells in the context of a degenerative phenomenon rather than true endocrine cell hyperplasia [10]. The mucinous epithelium often demonstrates morphologic features and immunophenotype suggestive of intestinal differentiation [11, 12]. As in our current case, the stromal endocrine cell micronests in previously reported cases demonstrated bland cytological features without atypia or mitotic activity. The endocrine cell micronests were immunoreactive for CK7, chromogranin, synaptophysin, and CD56 with negative reactions for CK20. The differential diagnosis in our case initially included sex cord stromal elements or microinvasion of the mucinous neoplasm [10, 13]. The focal continuity of endocrine cell clusters with adjacent mucinous glands and the presence of intraglandular endocrine cell proliferation provided useful diagnostic clues. Given the periglandular location with patchy distribution of endocrine cell micronests, the diagnosis of tumor microinvasion in the setting of a mucinous borderline tumor may be entertained; however, unlike microinvasion, endocrine cell micronests show classic neuroendocrine cytomorphology and are morphologically distinct from adjacent mucinous epithelium. Immunohistochemical studies with antibodies directed against neuroendocrine markers can aid in recognizing these endocrine cells when arranged in small solid nests with limited cell quantity and distinction from tumor microinvasion is not straightforward.

**Table 1:** Report of ovarian endocrine cell micronests: cases published between 1999 – present (including our case)

	Age/Sex	Ovarian tumor	Presenting symptom	Tumor size (cm)	Immunohistochemistry/Special stains		Follow-up (mo)
					**Positive**	**Negative**	
Ishikura et al., 1999 [7]	59/F	Ovarian mucinous cystadenofibroma	Abdominal pain	6	Chromogranin and keratin (weak); Stains for argentaffin cells (Fontana-Masson) and argyrophil cells (Grimelius)	Neurophysin, gastrin, insulin, glucagon, somatostatin	NED, 12 mo
Stewart et al., 2008 [8]	61/F	Ovarian mucinous adenocarcinoma with mucinous borderline component	Tiredness, abdominal discomfort, bloating	15	CK7, chromogranin, synaptophysin, CD56	CK20	Adjuvant chemotherapy, NED, 16 mo
Stewart et al., 2021 [10]	31/F	Ovarian mucinous cystadenoma	Incidental finding at screening ultrasound for pregnancy	16	CK7 (focal, strong), chromogranin and synaptophysin (diffuse, strong), CDX2 and PAX8 (focal, moderate)	CK20, ER, MUC5AC, MUC6, inhibin, calretinin, SF1	NR
Current case	27 F	Mucinous borderline tumor	Abdominal pain and fullness	23	CK, EMA, synaptophysin; Ki-67 proliferation index low (<1%)	Inhibin	NED, 10 mo

Abbreviations: *CD* cluster of differentiation, *CK* cytokeratin, *EMA* epithelial membrane antigen, *ER* estrogen receptor, *MUC5AC* mucin 5AC, *MUC6* mucin 6, *PAX8* paired box gene 8, *SF1* steroidogenic factor 1

Interestingly, the presence of stromal endocrine cell micronests and endocrine cell hyperplasia in adjacent mucinous glands, as in our case, are similar to those seen in autoimmune gastritis/chronic atrophic gastritis [14–16]. The majority of ovarian carcinoid tumors are associated with teratomatous components or by themselves, considered to be of germ cell origin. Cases of ovarian carcinoid tumors arising in the setting of ovarian mucinous neoplasms have been reported [17, 18]. The distinction between endocrine cell micronests and carcinoid/well-differentiated neuroendocrine tumor is somewhat subjective, but neuroendocrine tumors tend to be large, often with an expansile rather than infiltrative growth pattern and the tumor cells are arranged in insular, trabecular, or solid patterns with associated hyalinized, fibrotic stroma which are lacking with benign endocrine cell micronests [15].

An observation that is worth mentioning is the close spatial relationship between stromal endocrine cell micronests and mucinous glands with degeneration. As in our case, it is not uncommon to see neuroendocrine cells within reactive stroma with foamy histiocytic infiltration adjacent to degenerated mucinous epithelium. Our morphologic findings offer convincing evidence in support of preservation of endocrine cells in the setting of mucinous glandular degeneration as the most plausible underlying pathogenesis of stromal endocrine cell micronests [10]. The intraglandular endocrine cells are not evenly distributed in the mucinous epithelium and when present they may be arranged in single-cell fashion as well as aggregates that are clearly evident on routine H&E stain. These findings suggest that a background endocrine cell hyperplasia does exist and is similar to two previous reported cases [7, 8]. As mentioned earlier, the distinction between endocrine cell micronests and a neuroendocrine tumor can be difficult and sometimes arbitrary. There is no clear evidence of using Ki-67 labeling index as a prognostic marker in the setting of primary ovarian carcinoid tumors. However, Ki-67 labeling index may provide some useful information when metastatic neuroendocrine tumors from other organs are considered. These tumors often exhibit a higher Ki-67 labeling index compared to primary ovarian carcinoid tumors and portend a worse prognosis [19].

In summary, we report a mucinous borderline tumor with endocrine cell micronests that most likely represents preservation of endocrine cells in the setting of mucinous gland degeneration. Careful consideration of the histological and immunohistochemical features will help to avoid misinterpretation as microinvasion or sex cord stromal elements. Extensive sampling and a diligent search for any teratomatous components will exclude ovarian teratoma as a diagnostic consideration. We conclude that the presence of endocrine cell micronests are likely a benign morphological finding and although rarely reported, is a more common occurrence that is under-reported and should be interpreted as clinically insignificant when encountered.

## Data Availability

Not applicable.
